# Supplementary material for: Enhanced Extracellular Transfer of HLA-DQ Activates CD3+ Lymphocytes towards Compromised Treg Induction in Celiac Disease
Source: Int J Mol Sci. 2022 May 29;23(11):6102. doi: 10.3390/ijms23116102 (PMC9181181; doi:10.3390/ijms23116102)
Supplement: Supplementary file 1 [file ijms-23-06102-s001.zip › ijms-1724204-supplementary.pdf]

Celiac Disease	CeD
HLA Class II	HLA-II
Major Histocompatibility Complex	MHC
Monocyte Derived Dendritic Cells	MoDCs
Antigen Presenting Cell	APC
T-Cell Receptor	TCR
T-regulatory Cells	Tregs
Conditioned Growth Media	CGM
Healthy Controls	CTL
Lipopolysaccharide	LPS
Exosome Localization Motif	ELM
Complete media with inclusion of human granulocyte-macrophage colony-stimulating factor and human interleukin - 4	CMI
Complete media with inclusion of human granulocyte-macrophage colony-stimulating factor and human interleukin - 4 and exosome depleted foetal bovine serum	CMII
Curved Flow Cytometric Histograms	CFCHs
Mean Fluorescence Intensity	MFI
Non-conditioned media	NCM
Non-conditioned media with LPS stimulant	NCS
Leukocyte Function-associated Antigen-1	LFA-1
Tissue transglutaminase 2	tTGA
Gluten-free diet	GFD
Peripheral Blood Mononuclear Cells	PBMCs
Room Temperature	RT
Complete Medium: RPMI medium, FBS, antibiotic – antimycotic solution	CM
Granulocyte-Macrophage Colony-Stimulating Factor	GM-CSF
Immuno-Polymorphism Database – International Immunogenetics Project – Human Leukocyte Antigen	IPD-IMGT/HLA
Foetal Bovine Serum	FBS
Growth media	GM

Supplementary Table S1: List of abbreviations used throughout.

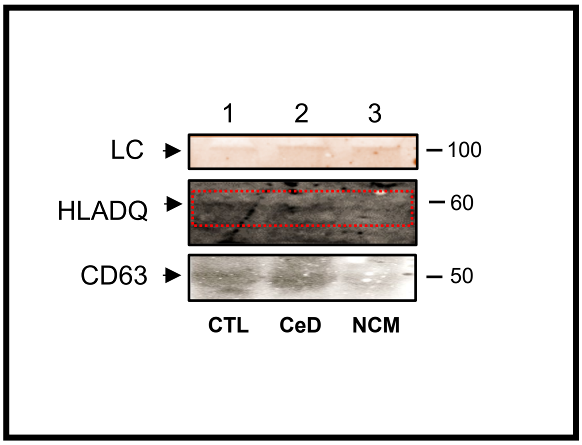

Supplementary Figure S1 (supporting Figure 3): Representative Western blots showing that HLADQ is expressed on exosomes. These were extracted from conditioned media exposed to monocyte derived dendritic cells of healthy control individuals (CTL), celiac disease patients (CeD) or non-conditioned media (NCM). HLADQ appears as a heterodimer given the molecular weight of approx.60 Kd. The presence of exosomes is indicated by CD63 staining. Nytran membrane was Ponceau S stained with a non-specific band representing a loading control in the secreted samples (LC).
